# Embedment of 3D Printed Self-Sensing Composites for Smart Cementitious Components

**DOI:** 10.3390/s25196005

**Published:** 2025-09-29

**Authors:** Han Liu, Israel Sousa, Simon Laflamme, Shelby E. Doyle, Antonella D’Alessandro, Filippo Ubertini

**Affiliations:** 1 Department of Civil, Construction, and Environmental Engineering, Iowa State University, Ames, IA 50010, USA; liuhan@iastate.edu; 2 Department of Civil and Environmental Engineering, University of Perugia, Via G. Duranti, 93, 06125 Perugia, Italyfilippo.ubertini@unipg.it (F.U.); 3 Department of Electrical and Computer Engineering, Iowa State University, Ames, IA 50010, USA; 4 Department of Architecture, Iowa State University, Ames, IA 50010, USA; doyle@iastate.edu

**Keywords:** 3D printing, cementitious composites, non-destructive evaluation, structural health monitoring, self-sensing, strain sensing, resistor, carbon microfiber

## Abstract

The automation of concrete constructions through 3D printing (3DP) has been increasingly developed and adopted in civil engineering due to its promising advantages over traditional construction methods. However, widespread implementation is hindered by uncertainties in quality control, homogeneity, and interlayer bonding, as well as the uniqueness of each printed component. Building upon our prior work in developing 3D-printable self-sensing cementitious materials by incorporating graphite powder and carbon microfibers into a cementitious matrix to enhance its piezoresistive properties, this study aims at enabling condition assessment of cementitious 3DP by integrating the self-sensing materials as sensing nodes within conventional components. Three different 3D-printed strip patterns, consisting of one, two, and three strip lines that mimic the pattern used in fabricating foil strain gauges were investigated as conductive electrode designs to impart strain sensing capabilities, and characterized from a series of quasi-static and dynamic tests. Results demonstrate that the three-strip design yielded the highest sensitivity ($\lambda_{\mathrm{stat}}$ of 669, $\lambda_{\mathrm{dyn}}$ of 630), whereas the two-strip design produced the highest signal quality (SNR_stat_ = 9.5 dB, SNR_dyn_ = 10.8 dB). These findings confirm the feasibility of integrating 3D-printed self-sensing cementitious materials through hybrid manufacturing, enabling monitoring of print quality, detection of load path changes, and identification of potential defects.

## 1. Introduction

Three-dimensional printing (3DP) technology leverages automated deposition processes for enhanced versatility, enabling precise control over material placement and facilitating the construction of structurally optimized and aesthetically complex architecture without the need for formwork and extensive manual intervention [1,2,3]. Three-dimensional printing of cementitious materials has the potential to transform the construction industry by reducing labor demands, accelerating construction speed, and enhancing sustainability through reduced material waste and use of friendly concrete mixes [4,5,6,7]. Yet, the widespread adoption and implementation of 3DP of cementitious components in civil engineering is impeded by the inherent variability introduced by the layer-by-layer additive manufacturing process, uncertainties in inter-layer adhesion, material homogeneity, and uncertain structural integrity of the printed components [8,9,10].

More specifically, layer adhesion variability is known to significantly influence structural integrity and mechanical performance, potentially leading to localized defects and compromising structural safety over the service life of printed structures [11,12,13,14]. The complex interaction between printing parameters, cementitious mix formulations, and environmental conditions introduces substantial uncertainties that are not readily detectable through conventional visual or manual inspection techniques [15,16,17]. Moreover, points of production interruption could be critical for a 3D-printed structure. For example, Wolfs et al. [11] measured a 16% reduction in flexural tensile strength and a 21% reduction in splitting tensile strength when the interlayer interval time increased from 15 s to 24 h. Skibicki et al. [14] reported a 56.6% decrease in interlayer bond strength after a 2 h pause (up to 68.5% at 4.5 h), accompanied by interfacial cracking and longitudinal pores. Advanced condition assessment methods capable of providing reliable, real-time feedback during and immediately after the printing process are critically needed to ensure consistent quality and structural reliability of 3D-printed concrete components [18,19].

Structural health monitoring (SHM) techniques present a viable approach to address such challenges by enabling real-time tracking of structural performance and early detection and identification of specific defects and/or performance degradation [20,21,22]. SHM methods are based on; for example, strain gauges [23,24,25], fiber optics [26,27,28], and piezoelectric transducers [29,30,31,32] including acoustic emission sensors [33,34,35], have demonstrated capabilities in monitoring structural behavior. Nevertheless, their application to 3D printed cementitious elements remains challenging due to integration concerns, application issues, maintenance, and difficult mapping between signal and structural conditions [36,37].

A dedicated SHM solution is to functionalize materials with strain-sensing capabilities, thus producing what is known as self-sensing materials [38,39,40]. By integrating self-sensing capabilities, the 3D printed components can directly provide real-time information on the quality of print via the interpretation of strain measurements [37]. This multi-functionality eliminates the need for external sensors, facilitating seamless integration into structural elements and simplifying sensor deployment and maintenance procedures [41,42,43]. These composites are typically fabricated by amplifying the materials’ piezo-resistive characteristics, allowing them to act simultaneously as structural elements and sensors capable of measuring strain through changes in electrical properties [44,45,46]. This can be done by incorporating conductive fillers such as carbon fibers [47,48,49], graphite (G) [50,51,52], carbon nanotubes [53,54,55], carbon black [56,57,58], and metal oxide [59,60,61] derivatives into the cementitious matrix. Compared to conventional SHM sensors, functionalized cementitious materials substantially improved mechanical robustness [62,63], enhanced durability [64], high compatibility [65], and reliability during the whole service life of the monitored structure [66]. The performance of these self-sensing materials has been demonstrated in the literature under various applications, including self-sensing pavements [67,68], railway sleepers [69], reinforced-concrete beams [70,71,72], footbridges [73], and masonry structures [74,75,76].

Our previous studies demonstrated that integrating G and carbon microfibers (CMF) into cementitious matrices could yield composites with enhanced piezoresistive responses to mechanical strain, allowing measurable changes in electrical resistance upon deformation [77]. Specifically, specimens doped with 10%wt G and 0.0032%wt CMF yielded an average gauge factor of 336 under compression, compared to a value of 36 for the plain specimens. We also demonstrated that we could 3D print self-sensing cementitious mixes using these dopants combined with cement paste [78] and ultra-high performance concrete [79]. The intent was to integrate self-sensing nodes within a 3DP cementitious element to empower condition assessment during the print and/or in operations. The integration of 3DP self-sensing nodes could be a viable solution to implement sensors over large 3DP scales, and to alleviate complications related to heterogenous mixes; for example, through the inclusion of aggregates (e.g., traditional concrete or supplementary cementitious materials). This study extends the prior research by studying the embedment of self-sensing cementitious materials within a traditional cementitious component to demonstrate the integration of self-sensing nodes, here creating smart cementitious blocks. We evaluate different self-sensing node designs to enhance strain-sensing accuracy, sensitivity, and robustness under mechanical loading conditions. Percolation curves are constructed to experimentally establish the optimal conductive cementitious mix. Afterwards, specimens are fabricated using three different 3D-printed zig-zag patterns, consisting of one-, two-, and three-strip lines, which mimic the pattern used in fabricating foil strain gauges. Note that the cementitious elements are made of traditional concrete and cast, and only the embedded self-sensing cementitious paste is 3D printed.

The novelty of this study is threefold, and consists of (1) embedding locally functionalized, extrusion-printed self-sensing nodes within conventional cementitious components, enabling condition assessment with minimal impact on the bulk material; (2) investigating three distinct 3D-printed zig-zag electrode patterns, inspired by foil strain gauges, to systematically evaluate the role of electrode geometry on sensing performance; and (3) experimentally demonstrating reliable and quantifiable strain sensing under both quasi-static and dynamic flexural loading. These contributions establish a feasible path to construct locally self-sensing structural materials through hybrid manufacturing.

The rest of the paper is organized as follows. Section 2 provides the background on 3D printed self-sensing cementitious specimens, including the material properties, the fabrication process, and the derivation of the electromechanical model. Section 3 describes the experimental methodology. Section 4 presents and discusses results from the experimental investigation. Section 5 concludes the paper.

## 2. Background

### 2.1. Carbon Microfibers (CMF)

Self-sensing cementitious materials typically consist of a cementitious matrix mixed with conductive fillers. The matrix acts as the binder and provides the structural integrity of the composite, while the conductive fillers introduce the electrical conductivity necessary to achieve percolation. In this study, the cementitious composite was formulated using G powder (Thermo Scientific Chemicals, Milwaukee, WI, USA, APS 7–11 μm, 99%), milled CMF (MCMF, SGL Carbon C M150-4.0/240-UN), and chopped CMF (CCMF, SGL Carbon C M150-4.0/240-G100), which collectively served as the primary dry materials for the 3D printing process. Both forms of CMF demonstrated excellent feedability, dispersibility, and compatibility with the cementitious material, and were thus used as received without further modification or purification. The selection of these materials was informed by prior studies showing that a hybrid incorporation of MCMF and CCMF effectively imparts self-sensing functionality to cementitious composites [77,78]. Both fiber types display smooth surfaces with occasional longitudinal grooves typical of PAN-based carbon fibers. A representative scanning electron microscopy (SEM) image of the CMF can be found in [77]. Detailed material properties are listed in Table 1. The parameter “single filament resistivity” refers to the intrinsic electrical resistivity of the carbon fiber material, as reported by the manufacturer (SGL Carbon GmbH, Arkadelphia, AR, USA). This value is independent of fiber length because both milled and chopped CMF originate from the same precursor and have identical diameters. For G, the reported value corresponds to the specific bulk resistivity of the powder material, as provided by the supplier, since graphite exists as flake-like particles rather than filaments.

**Figure 1:**
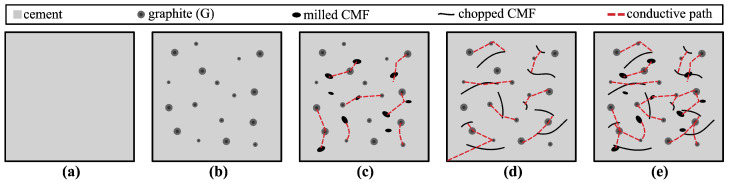
Illustration of the electrical percolation process: (**a**) cement-only (without fiber); (**b**) G-only; (**c**) G + MCMF; (**d**) G + CCMF; (**e**) G + MCMF + CCMF.

The electrical percolation mechanism of self-sensing cementitious composites is illustrated in Figure 1. In traditional cement-based materials, electrical conductivity is relatively limited and primarily governed by ionic conduction through pore solution and interconnected micro-pores (Figure 1a) [81]. To achieve enhanced electrical performance, conductive fillers are introduced to establish continuous electron-conducting networks. G is composed of carbon atoms arranged in hexagonal layers, possessing high electron mobility and electrical conductivity. G flakes tend to form conductive networks when dispersed in the cementitious matrix at sufficient volume fractions (Figure 1b). The proximity of particles is influenced by the packing density and degree of dispersion, both of which affect the ability of electrons to tunnel or hop between adjacent conductive regions [82]. Incorporating CMF can further enhance the connectivity of the conductive network. MCMF, characterized by shorter length and higher surface area, effectively interweaves with G flakes and fills microscopic gaps within the matrix (Figure 1c). CCMF, possessing a higher aspect ratio and elongated geometry, is capable of bridging larger voids, thereby accelerating the formation of long-range conductive pathways (Figure 1d).

However, higher-aspect-ratio fillers such as CCMF are more prone to agglomeration and entanglement, which can reduce the uniformity and signal repeatability [83,84]. In contrast, smaller aspect ratio fillers such as MCMF are less susceptible to reorientation or debonding during mixing and curing [85]. Dual-doping strategies that combine fillers with different aspect ratios have been widely reported in the literature for their synergistic effects on electrical and mechanical performance [58,86,87,88]. Specifically, the combination of MCMF and CCMF results in a multi-phasic and multi-scale conductive architecture in which MCMF occupies microscopic spaces between particles, while CCMF bridges macroscopic voids, together forming a more efficient and robust percolation network (Figure 1e). This hybrid network can accelerate electrical percolation and also improves sensitivity, repeatability, and structural performance of the functionalized cementitious composite sensors.

### 2.2. Composite Mixtures

**Figure 2:**
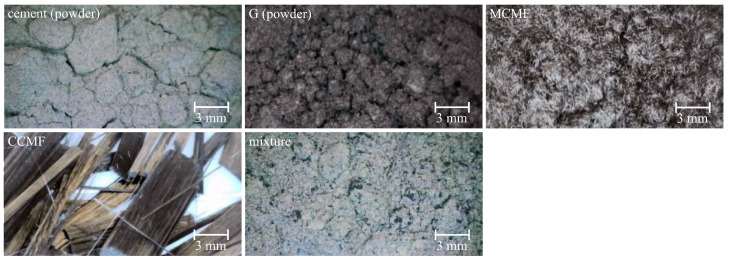
Magnified pictures of dry mixture components.

The self-sensing cement composite mixture used in this study combines commercially available Type IL Portland-limestone cement (64.18 wt% CaO, 20.09 wt% SiO_2_, 4.36 wt% Al_2_O_3_, 3.25 wt% Fe_2_O_3_, 2.01 wt% MgO, 0.68 wt% K_2_O, 0.23 wt% TiO_2_, and 0.1 wt% Na_2_O), G, MCMF, CCMF, and water. Figure 2 presents magnified images of these solid components, while their material properties, including density, conductivity, and aspect ratio, are listed in Table 2.

The mixture proportions for each set of specimens are summarized in Table 3, listing the material quantities in terms of weight, weight-to-cement ratio, and water-to-cement (w/c) ratios. Specimen labels begin with a numeric prefix indicating the weight percentage (wt%) of conductive filler relative to the weight of cement, followed by G, MCMF, or CCMF. G powder was first introduced at an initial concentration of 1.2 g (1 wt%) and then incrementally doubled up to 19.2 g (16 wt%) in order to determine the critical concentration for achieving electrical percolation. Based on these results, constant doping levels of 5 wt% and 10 wt% G were eventually selected, as they fall within the percolation zone and exhibit good workability and mechanical durability for the composite material in prior investigation [77,78].

For the 5 wt% G mixture, the MCMF content started at 0.15 g (0.125 wt%) and increased with a 0.15 g interval until reaching 0.6 g (0.5 wt%), while the CCMF content remained constant at 0.15 g (0.125 wt%). A similar approach was adopted for the 10 wt% G mixture, in which the G content was doubled to examine whether a higher baseline of conductive filler facilitated the formation of an electrical network. For these mixtures, the CCMF content was slightly increased (from 0.125 wt% to 0.167 wt%) to improve the dispersion of graphite and enhance conductivity by promoting better particle connectivity. The CCMF dosage was limited to 0.167 wt% in this study to prevent excessive viscosity and to minimize wear on the nozzle of the syringe, which could otherwise clog during extrusion.

The w/c ratios were set at 0.483 and 0.583 for 5 wt% G and 10 wt% G, respectively, across all mixtures. These values were selected based on our preliminary investigation, which showed that the w/c ratio needs to be increased with higher G powder content. This adjustment is attributed to the high surface area of the graphite particles, which absorb mixing water and thus require additional water to maintain sufficient workability, ensure homogeneous dispersion, retain adequate flowability for layer-by-layer extrusion, maintain adequate buildability for 3D printing, and support proper hydration of the mixture [89].

The w/c ratios were set at 0.483 and 0.583 for 5 wt% G and 10 wt% G, respectively, across all mixtures. These values were selected based on preliminary investigations, which showed that higher graphite contents require additional mixing water to offset the large surface area of the graphite particles and their strong tendency to absorb water. This adjustment ensured adequate flowability for layer-by-layer extrusion, buildability for 3D printing, and proper hydration of the mixture [89]. Beyond adjusting the water-to-cement ratio, the incorporation of graphite has broader implications for the rheological behavior and workability of fresh cementitious mixtures. Barbero-Barrera et al. [90] reported that the addition of 10 wt% G reduced the mini-slump flow of cement mortar by approximately 25% compared to the reference mix, demonstrating the filling effect of G within the matrix. Similarly, Papanikolaou et al. [91] observed that replacing cement with 5 to 15% fine G decreased paste flow diameters by 15 to 40%, with sharper reductions as particle fineness increased. Rehman et al. [92] noted that printable concretes require flow diameters in the range of 160 to 190 mm (mini-slump) to balance extrudability and buildability, and inadequate flowability leads to weak interlayer adhesion and poor surface quality. Maintaining sufficient workability is therefore essential to achieve continuous extrusion and homogeneous filler dispersion. Defects such as poor interlayer bonding, surface roughness, and irregular strip geometries could also occur, ultimately compromising sensing performance. Future work will include dedicated rheological characterization to quantitatively link mixture workability with print quality and electromechanical response.

A set of pure cement sample (0G0CMF) specimens was also prepared for benchmarking results. The piezoresistive behavior of each mixture type was characterized through a series of dynamic tests. Based on the outcomes of these evaluations, the optimal mixture type (10G250M167CCMF) was selected for embedment within drop-cast concrete beam specimens to serve as localized sensing nodes. The relevant results and analyses will be presented in Section 4.

### 2.3. Fabrication Process

**Figure 3:**
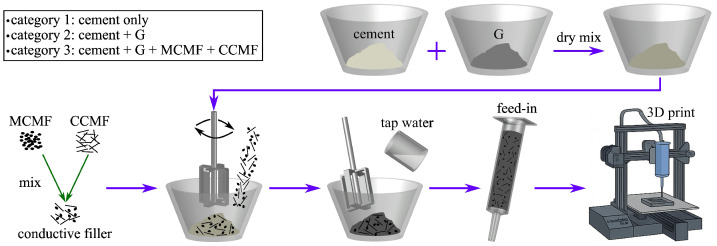
Fabrication process of the conductive cementitious composites.

A standardized mixing protocol was used here to prepare specimens and maintain consistent rheological properties of the composite mixtures [93], as presented in Figure 3. First, cement powder, G, MCMF, and CCMF were thoroughly mixed in their dry states with a spatula for approximately five minutes to break particle agglomerations. Next, 80% of the tap water by weight was added to the mixture and blended with a hand-held electric mixer at 200 rpm for two minutes. After, the remaining 20% water by weight was added to achieve the designed w/c ratios as listed in Table 3, and the mixture underwent additional blending for 3 min at 200 rpm to ensure homogeneity.

Using the framework developed in our prior work, an extrusion-based 3D printer (Creality Ender 3, Creality, Baytown, TX, USA) was modified by attaching a single-channel syringe pump (InfusionONE, New Era Pump Systems, Inc., Farmingdale, NY, USA) onto a custom support [78]. A 60 mL cylindrical syringe was installed on the pump to feed the composite mixture. The syringe measured 30 mm in diameter and 170 mm in length and was equipped with a nozzle featuring an inner diameter of 5.26 mm and an outer diameter of 7.52 mm. The inner surface of the syringe was lightly oiled prior to use to ensure continuous, uniform, and smooth extrusion of the mixture through the nozzle. The customized printer achieved a minimum resolution of 0.1 mm in the *x* and *y* axes, a 500 μm layer resolution in the *z* axis, a maximum printing speed of 20 mm/s, and a printable area of 235 × 235 mm^2^.

- Cube Specimens

**Figure 4:**
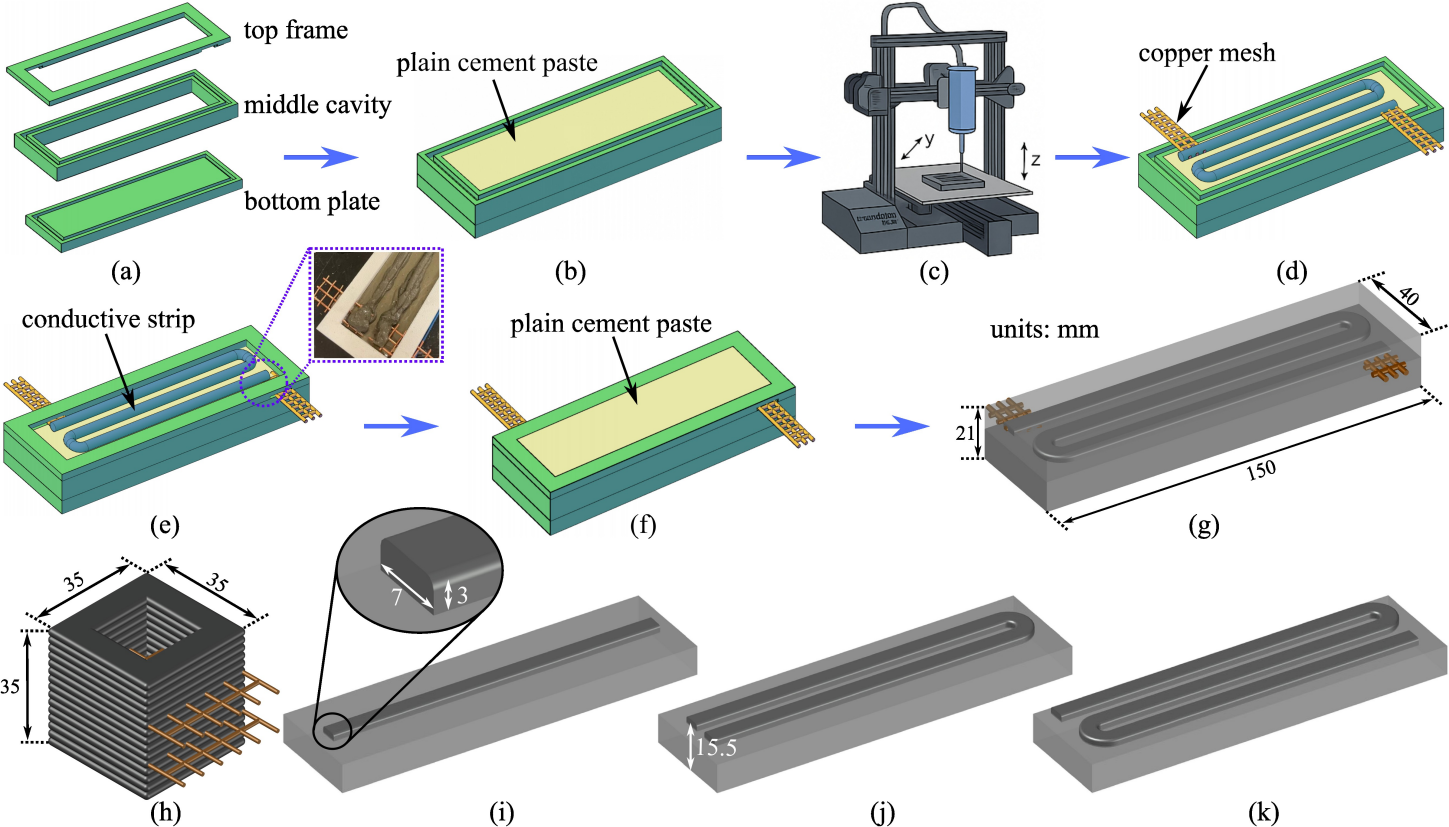
Fabrication process of the self-sensing cementitious specimen: (**a**) mold components; (**b**) drop-casting of the initial cementitious layer; (**c**) 3D printing of the self-sensing cementitious paste forming the conductive patterns; (**d**) embedment of conductive electrodes for external connections; (**e**) placement of the top part of the mold; (**f**) drop-casting of the top cementitious layer; (**g**) finalized specimen with annotated dimensions; (**h**) schematic showing the architecture and geometry of a 3D-printed conductive cementitious specimen; and (**i**–**k**) schematic drawings of the zig-zag patterns containing 1-, 2-, and 3-strip lines, respectively, with (**j**) schematic indicating the centroid of the printed strip at 15.5 mm from the bottom.

Cube specimens were 3D printed out of the conductive cement paste to establish the percolation curves. Hollow specimens of dimensions l×w×h = 35 × 35 × 35 mm^3^ were designed and sliced in Cura using 15 layers, each with a layer height of 2.33 mm, as shown in Figure 4h. The specimens were printed using a pump rate of 20 mm/min for an extrusion rate of 0.5 mm^3^/s, a printing speed of 8 mm/s, a layer width of 4.6 mm, and a stand-off distance of 3 mm (i.e., the gap between the nozzle tip and the build platform). An infill ratio of 0% was selected to create hollow geometries, resulting in an inner edge length of 24 mm. Prior to printing, an outer perimeter (or “skirt”) was deposited to purge air from the nozzle and ensure consistent and smooth material extrusion. A 120 s pause function was added in the G-code to create a 120 s pause at the fifth and tenth layers (heights of 11.65 mm and 23.33 mm, respectively) to allow manual insertion of two parallel copper meshes for embedding the sensing electrodes. During printing, a hot air gun (SEEKONE 1800 W) was positioned 150 mm from the specimen and set to 110 °C to accelerate curing and prevent slumping of freshly deposited layers. Sets of three cube specimens were fabricated for each mixture type, resulting in a total of 33 specimens.

- Small Beam Specimens

The beam specimens were fabricated through a laying process, as shown in Figure 4a–g. First, custom molds of overall dimensions l×w×h = 170 × 60 × 30 mm^3^ were fabricated, each constituted from three interlocking components: a top frame, a middle cavity block, and a bottom plate, as illustrated in Figure 4a. The top frame served as an alignment guide and defined the upper boundary of the beam. The middle section defines the space in which the composite material is cast and also provides space for positioning embedded electrodes. The bottom plate acts as the base and supports the shape during the curing process.

To fabricate the beam specimens, a Type IL Portland-limestone cement mixture with a water-to-cement (w/c) ratio of 0.40 was first prepared and drop-cast into the mold to a height of 15 mm. The mixture was allowed to rest for approximately 20 min to achieve initial setting. Next, the optimized self-sensing mixture was prepared using the standardized mixing procedure outlined earlier. The fully homogenized self-sensing mixture was manually loaded into the syringe, which was mounted onto the 3D printer. Following the same extrusion parameters used for fabricating the cube specimens, the self-sensing mixture was extruded into 1, 2, or 3 zig-zag strip lines, as shown in Figure 4i–k, using a pump rate of 20 mm/min, a printing speed of 8 mm/s, and a stand-off distance of 3 mm. The printed conductive strip was printed 14 mm from the bottom of the beam and was approximately 3 mm in height and 7 mm in width due to the lateral spreading of the extruded material upon deposition and the compaction caused by the nozzle, as shown in the onset of Figure 4i. Therefore, the centroid of the strip is located at 14 + 1.5 = 15.5 mm from the bottom surface, as indicated in Figure 4j. The zig-zag pattern was adopted to mimic the configuration of foil strain gauges, maximizing the length of conductive material subjected to strain in the parallel direction. Two copper meshes with a 5.15 mm opening size and 1.2 mm wire diameter, each spanning 120 mm, were embedded within the printed layer, forming a mechanical connection to the data acquisition system for resistance measurements. Lastly, the specimens were let to rest for 10 min, and a 6 mm layer of plain cement mixture with 0.40 w/c was prepared and drop-cast over the 3D-printed layer, as shown in Figure 4f. This process yielded a finished specimen with overall dimensions of l×w×h = 150 × 40 × 21 mm^3^, corresponding to the inner dimension of the mold, as shown in Figure 4g. Sets of four beam specimens were prepared with 1, 2, or 3 printed strip lines, yielding a total of 12 beam specimens.

Upon completion of printing, cube and beam specimens were covered with plastic sheeting to prevent rapid water evaporation and minimize shrinkage. Initial curing was performed under laboratory conditions at a constant temperature of 25 °C for 48 h. Subsequently, the specimens were transferred to a standard moisture-curing room maintained at 23 ± 2 °C and 95 ± 3% relative humidity for an additional 26 days, resulting in a total curing period of 28 days, consistent with standard concrete curing protocols. Prior to electrical and piezoresistive testing, all specimens were oven-dried at 60 °C for 24 h to eliminate excessive moisture, which could otherwise interfere with the accuracy of electrical resistivity measurements.

### 2.4. Electromechanical Model

The strain-sensing capability of a self-sensing cementitious material mostly stems from the material’s piezoresistive effect. Assuming only the internal resistance is influenced by the mechanical deformation [94,95], the conductive cementitious composite can be modeled as a resistor of nominal electric resistance $R_{0}$,

(1) $$R_{0}=\rho \frac{d}{A},$$

where ρ is the resistivity, *d* is the distance between the electrodes, and *A* is the cross-section area. Assuming small deformation, the fractional change in resistance (FCR) is taken as

(2) $$\mathrm{FCR}=\frac{\Delta R}{R_{0}}=\frac{R-R_{0}}{R_{0}},$$

where ΔR is the variation in resistance, and *R* is the resistance measured in real-time. For a beam subjected to bending, the maximum bending strain $\varepsilon_{\max}$ at the extreme fibers (top and bottom surfaces of the beam) is related to the deflection δ measured at the midpoint during a three-point bending test and is expressed as

(3) $$\varepsilon_{\max}=\frac{6\;\cdot \;\delta \;\cdot \;h}{L^{2}},$$

where δ represents the midpoint deflection, taken as the maximum displacement recorded by the testing machine; *h* is the height (or depth) of the beam; and *L* is the span length between the two supports. Assuming a linear strain distribution across the depth of the cross-section, with maximum strain occurring at the extreme fibers and zero strain at the neutral axis taken approximated at mid-height, the strain at distance (*y*) from the neutral axis is

(4) $$\varepsilon \left(y\right)=\frac{\varepsilon_{\max}}{h/2}\;\cdot \;y.$$

The bending strain at the location of the integrated sensing nodes, denoted ε(y), positioned 15.5 mm from the center of the conductive strip toward the base layer of the specimen with an overall height of 21 mm, as shown in Figure 4j, is evaluated at a distance y=15.5−10.5=5 mm from the neutral axis and expressed as

(5) $$\varepsilon \left(y=5\right)=\frac{5\;\cdot \;\varepsilon_{\max}}{h/2}=\frac{5\times 6\;\cdot \;\delta \;\cdot \;h}{10.5L^{2}}=\frac{20\;\cdot \;\delta \;\cdot \;h}{7L^{2}}.$$

The strain measured by the sensing material is the average strain along the length of the beam, approximated by taking half of the maximum strain ε(y=5) given the linearity of the bending moment

(6) $$\varepsilon_{\mathrm{avg}}=\frac{1}{2}\;\cdot \;\left(\varepsilon \left(y=5\right)+0\right)=\frac{10\;\cdot \;\delta \;\cdot \;h}{7L^{2}}.$$

Note that the resistance measurement is taken between two electrodes spaced 120 mm apart, which approximately matches the span length used in the three-point bending setup. Therefore, no additional spatial averaging or reduction factor is used in Equation (6). By differentiating the natural logarithm of Equation (1), the FCR can be expressed as a function of the average bending strain $\varepsilon_{\mathrm{avg}}$,

(7) $$\mathrm{FCR}=\frac{\Delta \rho }{\rho }+\frac{\Delta d}{d}-\frac{\Delta A}{A}=\frac{\Delta \rho }{\rho }+\left(1+2\nu \right)\varepsilon_{\mathrm{avg}},$$

where ν is the Poisson’s ratio of the material, and Δρ/ρ is the piezoresistive effect that often dominates when at electrical percolation. The gauge factor $\lambda_{\mathrm{bend}}$ of the conductive cement composite under bending can be written as

(8) $$\lambda_{\mathrm{bend}}=\frac{\mathrm{FCR}}{\varepsilon_{\mathrm{avg}}}=\left(1+2\nu \right)+\frac{\Delta \rho }{\rho }\frac{1}{\varepsilon_{\mathrm{avg}}}.$$

The signal-to-noise ratio (${\mathrm{SNR}}_{dB}$) and mean absolute error (MAE) are utilized to quantitatively assess the signal quality, and are taken as

(9) $${\mathrm{SNR}}_{dB}=10\;\cdot \;\log_{10}\left(\frac{P_{\mathrm{signal}}}{P_{\mathrm{noise}}}\right),$$

(10) $$\mathrm{MAE}=\frac{\sum_{i=1}^{z}\left|x_{{\mathrm{true}}_{i}}-x_{{\mathrm{est}}_{i}}\right|}{z},$$

where ${\mathrm{SNR}}_{dB}$ is in decibels, $P_{\mathrm{signal}}$ and $P_{\mathrm{noise}}$ are the power of signal and noise, respectively, $x_{{\mathrm{true}}_{i}}$ and $x_{{\mathrm{est}}_{i}}$ are the true and measured values, respectively, and *z* is the total number of samples collected.

## 3. Experiments

### 3.1. Measurement

Percolation curves were obtained based on resistance measurements performed at 28 days of curing using a two-probe alternating current (AC) method. This AC approach was selected due to the anticipated high electrical resistivity and low conductivity of the specimens [96,97]. Electrical resistance was measured using an Agilent 4263B LCR meter (Agilent Technologies, Santa Clara, CA, USA), operated in resistance–reactance (R–X) mode at a frequency of 1 kHz. The system was driven through LabVIEW to monitor intrinsic polarization drift, which may arise from either the dielectric behavior of cement-based materials [98,99] or direct piezoelectric behavior [100,101]. The frequency of 1 kHz was selected to minimize dielectric interference and reduce complications due to electrode polarization and reactance [96,102]. The use of embedded copper meshes as electrodes lowered contact resistance by enhancing interfacial electrical connectivity and ensuring more uniform current distribution [103].

To evaluate the dispersion quality of conductive fillers, frequency response analysis was performed by measuring impedance over a frequency range from 100 Hz to 100 kHz. This analysis captured the frequency-dependent behavior of the internal conductive network. A stable impedance response across the frequency spectrum was considered indicative of uniform filler dispersion, whereas variations suggested issues such as particle agglomeration or uneven distribution [96,104]. Specimens displaying unstable signal behavior, impedance values outside an acceptable range, or significant resistance deviations compared to others within the same category were considered as inadequately dispersed and were excluded from further analysis and re-fabricated as necessary. Both compression and bending tests were carried out in order to evaluate the sensitivity in different conditions. Compression tests were performed on cubes with different mix designs to evaluate the best performing. Bending tests were implemented on the optimal mix with different configurations to evaluate the effects of the different setups and applications for structural elements.

### 3.2. Compression and Flexural Tests

**Figure 5:**
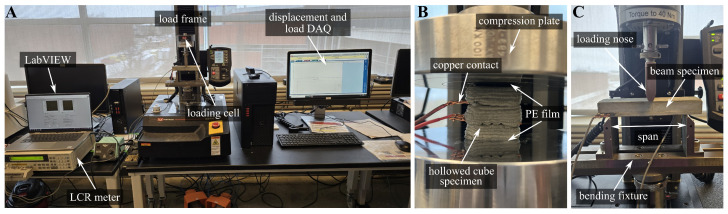
(**A**) Overall experimental configuration; (**B**) close-up view of the compression loading setup; and (**C**) close-up view of the flexural bending setup.

The strain-sensing performance of the cube specimens was evaluated through axial compression tests conducted 28 days after casting, following a procedure similar to that outlined in [78]. Figure 5A shows the overall experimental setup. The compression tests were carried out using an Instron 5944 universal testing machine equipped with a 1 kN 2580 series load cell. This load frame offers a measurement precision of ±0.01 mm and ±0.05 N and has been previously calibrated and validated for strain-sensing applications. Specimens were placed between two compression platens to ensure uniform load distribution, and the top and bottom surfaces of each specimen were lightly abraded prior to testing to provide smooth contact interfaces with the loading apparatus and to minimize any uneven load transfer. Two polyethylene (PE) films were placed on both the top and bottom sides of the specimen, between the specimen and the loading platen, to provide electrical insulation, as shown in Figure 5B. Due to the small size of the specimens and the direct axial loading configuration, it was assumed that the strain or force applied by the testing machine was that experienced by the materials. Prior to dynamic loading, a 50 N preload was applied to eliminate potential seating or settlement effects, yielding a specimen gauge length of approximately 35.3 to 36.2 mm. The dynamic loading protocol included five total cycles, and the load magnitudes for each cycle were set to 100, 200, 300, 400, and 500 N, with a constant loading frequency of 0.16 Hz, corresponding to the loading rate of 33.3, 66.6, 99.9, 133.3, and 166.6 N/s, respectively.

Similarly, the strain-sensing performance of the beam specimens was evaluated through three-point bending tests, including quasi-static and dynamic protocols. The closeup view of the experimental setup is shown in Figure 5C. Beam specimens of dimensions of l×w×h = 150 × 40 × 21 mm^3^ were placed horizontally on two supports spaced 120 mm apart along its longitudinal direction, complying with the configuration prescribed by ASTM C293. Prior to the quasi-static tests, a 120 s resistance measurement was conducted on each beam specimen without any applied load to characterize the intrinsic polarization drift. The quasi-static tests were designed to assess both the strain sensitivity and electrical noise characteristics under flexural loading. A preload of 20 N was applied to eliminate initial settlement effects, and a uniaxial concentric load was applied vertically at the mid-span of the specimen using a loading nose, with a constant loading rate of 0.2 mm/min. The loading was terminated when the applied force reached 300 N, yielding an average bending strain $\varepsilon_{\mathrm{ave}}$ (Equation (6)) of approximately 400 μϵ, in order to prevent specimens from cracking [105].

The dynamic tests were performed on the beam specimens to assess the repeatability and signal stability under cyclic loading. Before the dynamic sequence began, a preload of 10 N was also applied to minimize initial deformation and ensure consistent contact between the specimen and fixture. Tests were performed by applying triangular waveform dynamic loading across nine total cycles at a constant loading frequency of 0.2 Hz. The first three cycles reached a peak load of 100 N at a loading rate of 40 N/s, the next three cycles reached 200 N at 80 N/s, and the final three cycles peaked at 300 N with a loading rate of 160 N/s, matching the maximum load applied during the quasi-static test.

For both the compression and flexural tests, load and displacement data were recorded at a sampling rate of 100 Sample/second (S/s), and resistance measurements were simultaneously collected at 10 S/s using the LCR meter operated in the R-X mode at a frequency of 1 kHz. All tests were conducted in the laboratory under constant temperature conditions, and at least three specimens were tested for each mix design, with additional specimens tested as needed to ensure the consistency and reliability of the data.

## 4. Results and Discussion

### 4.1. Self-Sensing Cube Specimen

- Percolation

**Figure 6:**
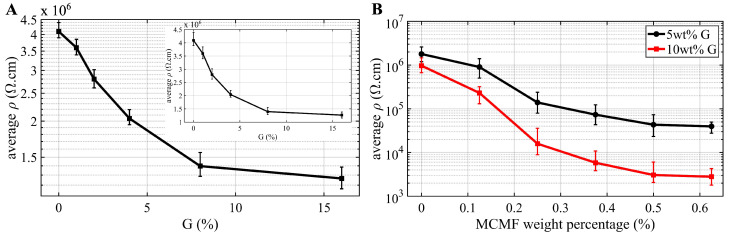
Percolation curves obtained from 28 to day resistivity measurements for (**A**) G filling with the inset showing the same plot in linear scale; and (**B**) dual doping (MCMF + CCMF) with different MCMF doping levels under 5 wt% and 10 wt% G filling.

Figure 6A present the 28-day three-specimen averaged resistivity (ρ) computed using Equation (1) as a function of G content on a semi-logarithmic scale, with error bars representing the minimum-to-maximum range of the resistivity measured over three specimens. For reference, a linear-scale version of the same plot is provided in the inset of Figure 6A. It can be observed that the resistivity of the self-sensing cement specimen decreases significantly with increasing G content, particularly in the lower range from 0 wt% to 8 wt%. Following these results, G dosages of 5 wt% and 10 wt% were selected for subsequent specimen fabrication.

The percolation behavior for specimens incorporating MCMF with 5 wt% and 10 wt% G is compared in Figure 6B. In these mixtures, CCMF content was fixed at 0.125 wt% for the 5 wt% G group and at 0.167 wt% for the 10 wt% G group. It can be observed that the percolation behavior of 5 wt% G (black line) and 10 wt% G (red line) follows a similar trend by exhibiting a noticeable decrease in resistivity at low concentrations from 0.125 wt% to 0.375 wt%, causing a reduction in resistivity of 70% and 80%, respectively. This can be explained by the reduced inter-fiber distance that facilitates more efficient electronic transition. Specimens with 10 wt% G consistently exhibit lower resistivity than those with 5 wt% G, attributed to the enhanced conductivity and accelerated percolation provided by the higher G content.

- Strain Sensing

**Figure 7:**
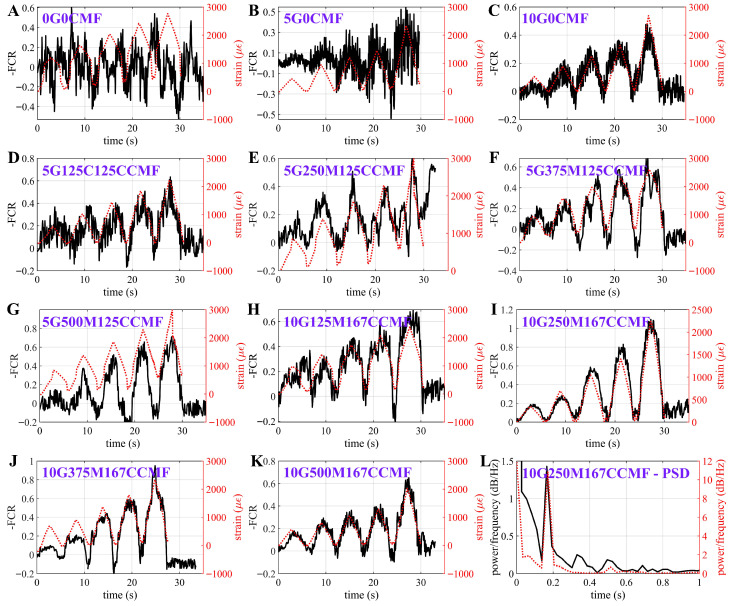
Time histories of electrical and strain measurements under cyclic loading for specimens (**A**) 0G0CMF; (**B**) 5G0CMF; (**C**) 10G0CMF; (**D**) 5G125M125CCMF; (**E**) 5G250M125CCMF; (**F**) 5G375M125CCMF; (**G**) 5G500M125CCMF; (**H**) 10G125M167CCMF; (**I**) 10G250M167CCMF; (**J**) 10G375M167CCMF; (**K**) 10G500M167CCMF; and (**L**) PSD plot for the 10G250M167CCMF specimen.

Figure 7A–K presents the electrical signal compared against the strain input extracted from the Instron machine for representative specimens from each mix design. Presented datasets are taken from the best-performing specimen under each mixture type in terms of the gauge factor $\lambda_{\mathrm{dyn}}$, which was obtained using the average power spectral densities (PSD) of the cyclic loading data and computed as the ratio of the resistance-to-strain changes. An example PSD plot of the electrical response from the 10G250M167CCMF specimen, along with its corresponding strain input under a 0.2 Hz dynamic excitation, is shown in Figure 7L. It can be observed that the cement-only specimen (0G0CMF) failed to provide meaningful measurements (Figure 7A), indicating that the response is primarily governed by ionic conduction, rather than a governing piezoresistive mechanism [39]. The inclusion of G notably enhanced sensing performance, as demonstrated by the improved correlation between the electrical signal and strain time histories. In particular, specimens doped with 10 wt% G (Figure 7C) exhibited significantly reduced signal noise and improved strain tracking capability compared to those with 5 wt% G (Figure 7B), attributable to the formation of a more robust and saturated conductive network at higher G content, which minimizes local impedance fluctuations and enhances the stability of electron transport paths under loading. Moreover, specimens incorporating hybrid MCMF + CCMF fillers (Figure 7D–K) demonstrated further enhancements in strain-sensing performance through the increase in FCR magnitudes and signal-strain correlations, indicating improved strain sensitivity and a substantial reduction in signal noise compared to their single G-doped counterparts (5G0CMF and 10G0CMF).

- Sensing Performance Metrics

**Figure 8:**
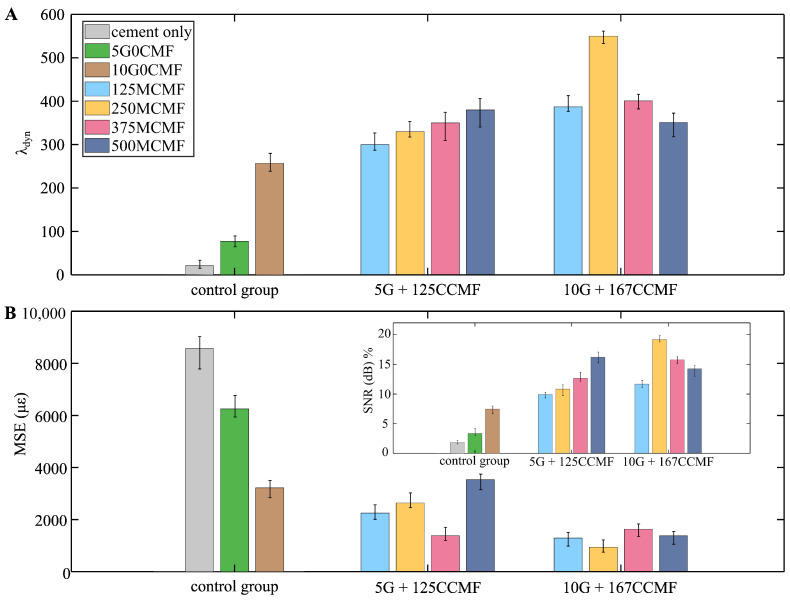
Bar chart of three-specimen-averaged sensing metrics for the cube specimens: (**A**) dynamic gauge factor ($\lambda_{\mathrm{dyn}}$); and (**B**) MAE with the onset showing the SNR, comparing different mixture types.

Figure 8A,B are the bar charts comparing the three-sample averaged dynamic gauge factor ($\lambda_{\mathrm{dyn}}$), SNR (onset of Figure 8B), and MAE, respectively, with the error bars indicating the full range across the three specimens in each category. A distinct spectral peak at the excitation frequency is clearly observed above noise, demonstrating strong signal clarity and frequency correlation between electrical and mechanical responses. The SNR and MAE were computed using Equations (9) and (10), respectively. The SNR is expressed in decibels relative to the carrier (dB) for a real-valued sinusoidal input, serving as a quantitative measure of signal quality by comparing the peak signal to the background noise level. The MAE quantifies the average absolute deviation between the resistance response derived strain and the reference strain input, providing an indication of tracking accuracy.

Table 4 assembles the quantitative results taken from Figure 8 along with the percentage increment (Δ) computed with respect to the 5 wt% G-doped specimen (i.e., 5G0CMF) for $\lambda_{\mathrm{dyn}}$, SNR, and MAE. It can be observed that specimens doped with CMF consistently outperform cement-only and G-only specimens, exhibiting higher $\lambda_{\mathrm{dyn}}$ and SNR values. In contrast, the MAE followed an inverse trend, with lower values indicating improved tracking accuracy. These improvements are attributed to the enhanced electrical connectivity achieved through the formation of multi-scale, interconnected conductive networks within the hybrid matrix. The addition of both MCMF and CCMF bridges micro- and macro-scale gaps between graphite flakes, effectively reducing signal attenuation and enhancing sensitivity. This hybrid conductive framework enables more efficient charge transport under strain and improves the signal-to-noise performance by minimizing discontinuities and dispersion irregularities.

One can also observe that inclusion of higher graphite content (10 wt% G) and increased CCMF dosage (0.167 wt%) generally yields superior sensing performance compared to mixtures with 5 wt% G and 0.125 wt% CCMF when evaluated at the same MCMF inclusion level. An exception to this trend is the 10G500M167CCMF specimen, which appears to be saturated with conductive particles. In this case, excessive filler content may have led to filler agglomeration or short-circuiting within the matrix, thereby diminishing the piezoresistive sensitivity and reducing the SNR performance. From these results, hybrid mixture 10G250M167CCMF appears to be outperforming other mixtures in terms of the overall sensing performance, yielding the highest $\lambda_{\mathrm{dyn}}$ of 550, an SNR of 19.19, and the lowest MAE of 721, corresponding to net improvements of 480%, 473%, and 86%, respectively, when compared to the 10G0CMF specimen. Mixture 10G250M167CCMF was selected for fabricating the embedded conductive paste that served as localized sensing nodes in the beam specimen. Reported results represent the average over three specimens, with error bars in Figure 8 showing the specimen-to-specimen variability. The standard deviations and coefficients of variation (generally below 10%) confirm the reproducibility of the results. The observed improvements in sensing performance for hybrid-doped mixtures are consistent across specimens, thereby supporting the synergistic effect of combining G and CMF.

The sensing performance metrics for the 0G0MCMF mixture exhibit $\lambda_{\mathrm{dyn}}$ values nearly identical to those from prior studies [78], reported as 21. The 10G0CMF specimen resulted in a 148% higher $\lambda_{\mathrm{dyn}}$ than reported, also in [78], as 73. This discrepancy in performance is likely attributed to differences in the graphite particle properties used. Specifically, the G used in this study was sourced from Fisher Chemical (APS 7–11 μm), which differs from the Fisher Chemical G67-500 G powder used in prior work. The G67-500 grade consists of coarser particles with a broader size distribution. These differences in particle size, surface morphology, and potentially purity are critical factors that can significantly affect dispersion quality, the formation of conductive networks, and ultimately the piezoresistive response of the composite.

### 4.2. Self-Sensing Beam Specimen

- Quasi-static Sensing

**Figure 9:**
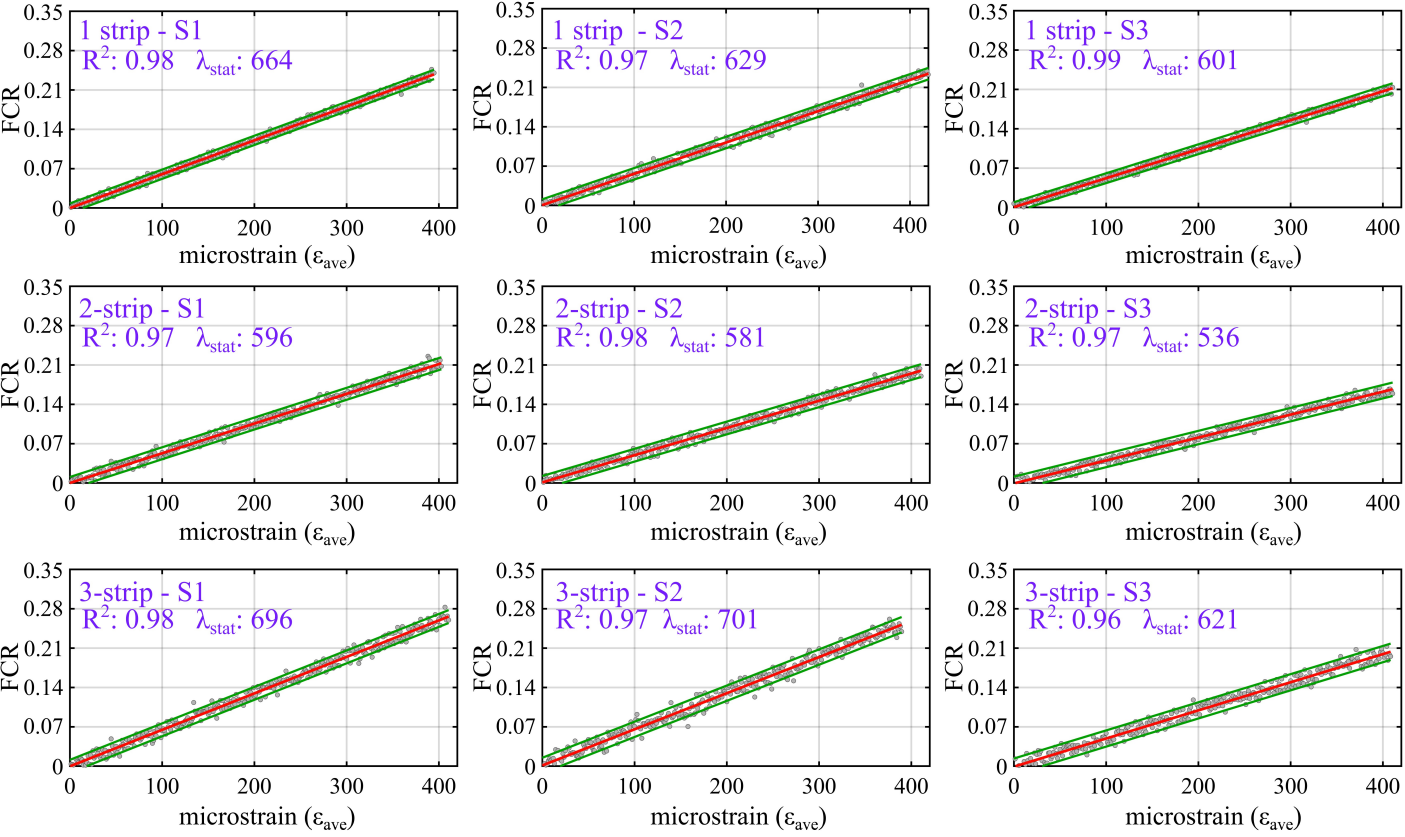
Plots of FCR measured from the quasi-static test for each specimen with different 1-, 2-, and 3 strip configurations, showing the linear fit (red) and 95% confidence interval (green).

Figure 9 plots the relationship between FCR (Equation (2)) and the average bending strain ($\varepsilon_{\mathrm{ave}}$), computed based on Equation (6). Results include a linear regression fit (solid red line) along with its corresponding 95% confidence interval (CI) bounds (dashed green lines). The coefficient of determination (*R*^2^), which quantifies the linearity of the response, and the static gauge factor ($\lambda_{\mathrm{stat}}$), obtained as the slope of the fitted line, are reported within each subplot. Data are presented post-processed by using a Savitzky–Golay filter with a polynomial order of 1 and a window size of 100 to remove the baseline drift attributed to intrinsic polarization effects, which could otherwise distort the linear trend of the FCR response. This window size was selected based on the sampling rate (10 S/s) and the loading frequency (0.2 Hz), corresponding to approximately half of one cycle of dynamic loading. This choice provided effective smoothing while retaining the amplitude and phase of the cyclic strain response. Trial comparisons with window sizes of 70 and 130 yielded differences in peak amplitude and gauge factor of less than 5%, confirming that the selected window size did not alter the true strain fluctuations. Table 5 compiles the sensing metrics of each of the three specimens (S1–S3), extracted from Figure 9, embedded with one-, two-, and three-line patterns along with their averaged value. Reported values include the static signal-to-noise ratio (SNR_stat_), *R*^2^, and $\lambda_{\mathrm{stat}}$. The resolution is defined as the range of FCR values within the 95% CI and the corresponding strain levels, computed using Equation (8). Measurement accuracy, denoted as $\sigma_{\mathrm{res}}$, is quantified by the standard deviation of the resolution measured on the three specimens.

The results show that all beam specimens exhibited a good linearity in the quasi-static tests by yielding the averaged *R*^2^ value between 0.97 and 0.98 across the three configurations, indicating an excellent linear correlation between strain and electrical signal. Among them, the two-strip design demonstrated the highest signal quality by resulting in an average static SNR_stat_ of 9.50 dB. In comparison, the one-strip and three-strip configurations yielded slightly lower static SNR values of 8.58 dB and 7.98 dB, respectively. In terms of sensitivity, the three-strip configuration exhibited the highest average static gauge factor ($\lambda_{\mathrm{stat}}$) of 669, followed by one-strip and two-strip, with values of 631 and 571, respectively; while all designs demonstrated reliable linearity and signal clarity, notable differences were observed in terms of resolution and accuracy. The one-strip configuration achieved the lowest equivalent strain resolution of 34 μϵ, followed by the two-strip and three-strip groups with resolutions of 44 μϵ and 46 μϵ, respectively. This finding suggests that the one-strip design is more sensitive to small strain variations, attributable to the averaging effect of the higher number of strips. The corresponding sensing accuracy ($\sigma_{\mathrm{res}}$), computed as the standard deviation of the resolution, shows that the one-strip and two-strip configurations achieved the highest repeatability, outperforming the three-strip configuration at 10.01 μϵ.

- Dynamic Sensing

**Figure 10:**
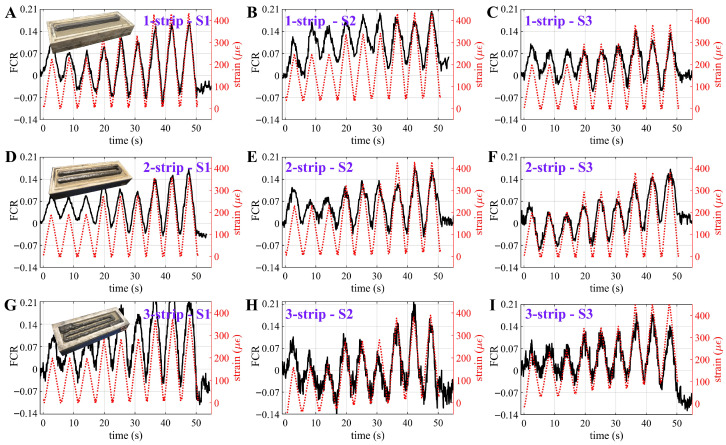
Time-series plots of the FCR measured from each beam specimen under flexural bending with (**A**–**C**) 1; (**D**–**F**) 2; and (**G**–**I**) 3 conductive strips, respectively.

Figure 10A–I present time-series plots of the FCR of each of the three sets of specimens embedded with one-, two-, and three-strip configurations, respectively, compared against the average bending strain ($\varepsilon_{\mathrm{avg}}$) computed from Equation (6), during the dynamic test. Relevant pictures of the specimens with one-, two-, and three-strip configurations are provided in the insets. The data were post-processed using a Savitzky–Golay filter as for the static tests (Figure 9). The results show a close match between the electrical signal and strain time histories, indicating that the beam specimen is capable of tracking the strain input. Table 5 summarizes the key dynamic strain-sensing performance, including dynamic SNR_dyn_, MAE, and $\lambda_{\mathrm{dyn}}$. In terms of the three-specimen averaged value, it can be found that the one-strip configuration yielded the highest MAE of 228 μϵ and a dynamic SNR_dyn_ of 8.58 dB. The two-strip configuration exhibited a comparable MAE of 198 μϵ but the highest SNR_dyn_ of 9.50 dB. The three-strip configuration exhibited the lowest average MAE of 185 μϵ, yet also the lowest SNR_dyn_ of 7.98 dB. Despite its slightly reduced signal clarity, the three-strip configuration demonstrated the highest $\lambda_{\mathrm{dyn}}$ of 630, compared to 583 for the one-strip and 510 for the two-strip.

- Sensing Performance Metrics

The differences in the dynamic gauge factor $\lambda_{\mathrm{dyn}}$ across configurations are not very substantial, with values of 583, 510, and 630 (mean = 574.3, standard deviation = 60.5, and Coefficient of variation = 10.5%), as it should be expected (Equation (8)). The differences between $\lambda_{\mathrm{dyn}}$ across specimens can be attributed to the heterogeneity caused by hand-processed materials and fabrication steps that can create unwanted nonlinear behaviors and localized, more sensitive responses to strain having higher chances of being experienced by a three-strip configuration that spans a larger surface. This can further explain the higher standard deviation $\sigma_{\mathrm{res}}$, lower overall MAE, and lower overall SNR. The two-strip configuration yielded the lowest $\lambda_{\mathrm{dyn}}$, but the sensing element does not cover the center line of the beam unlike the two other configurations. The overall lower values for $\lambda_{\mathrm{dyn}}$ compared to $\lambda_{\mathrm{stat}}$ are an artifact of the frequency dependence and is consistent with literature [106]. At higher loading rates, mechanisms such as viscoelastic damping [107], interfacial slippage [108], and capacitive charge accumulation [109] can inhibit the full reconfiguration of conductive paths, thereby attenuating the measurable resistance change. Additionally, tunneling and contact resistance at fiber or flake junctions may not respond instantaneously to strain oscillations, especially under rapid cyclic deformation [39,110,111]. These time-dependent effects result in a lower apparent $\Delta R/R_{0}$, and thus a reduced gauge factor under dynamic excitation.

It can also be observed in Table 5 that the two-strip configuration outperformed the other two designs in terms of SNR. The more stable signal is likely attributable to the strips not covering the centerline of the beam. The average SNR_stat_ of the two-strip configuration was 10.7% higher than that of the one-strip, and 19.1% higher than the three-strip. Similarly, SNR_dyn_ was improved by 16.9% and 20.5% compared to the one-strip and three-strip configurations, respectively. These results suggest that a more automated fabrication process may improve on sensing performance through by reducing heterogenuities and thus the signals’ nonlinearities and the presence of localized strain in the sensing strip.

The resolution of specimens is also adequate and consistent with values obtained in prior work [78]. It is anticipated that the resolution can dramatically improve through the development of a more automated fabrication process and a dedicated data actuation system. These results, combined with high *R*^2^ values, demonstrate that it is possible to embed strain-sensitive nodes within traditional cementitious components. In practice, these nodes could be integrated through a hybrid manufacturing process; for example, by using multi-nozzle systems or by co-extrusion nozzles capable of depositing concentric or layered filaments. Vertical sensing paths, spanning multiple horizontal layers, could be created by strategically designing the location of conductive paste deposition. These approaches would allow three-dimensional sensor networks, enabling not only horizontal but also vertical monitoring of strain fields within large-scale printed components.

Results also show that the configuration of electrodes may have a role in the sensing performance. Overall, these bending results demonstrate that all three configurations exhibit good applicability, and their sensing characteristics could be tailored to meet the requirements of specific structural applications, such as bridges, buildings, or prefabricated concrete components where distributed strain monitoring under service loads and seismic excitations is required. However, further investigations would be required to further evaluate the impact of configurations, in particular on more realistic sizes. At this stage, results show that a three-strip configuration leads to a more sensitive sensor, yet with noisier signal, while the two-strip configuration leads to a more stable signal yet less strain-sensitive measurements, and the one-strip configuration exhibits better resolution.

## 5. Conclusions

This study investigated the performance of self-sensing cementitious materials fabricated by embedding 3D-printed self-sensing cementitious composites, with the intent to enable condition assessment of additively manufactured components. The self-sensing composite consisted of a cement mix doped with G and CMF used to reach electrical percolation and promote a high piezoresistive effect. A standardized fabrication protocol was established for the preparation of both self-sensing cube specimens and beam specimens functionalized with embedded sensing nodes.

The study first evaluated electrical percolation curves obtained by measuring the resistivity of different self-sensing cementitious mixes fabricated with different levels of CMF inclusions. Among the tested mixtures, the hybrid composition of 10G250M167CCMF exhibited superior performance, achieving a dynamic gauge factor ($\lambda_{\mathrm{dyn}}$) of 550, SNR of 19.19 dB, and MAE of 721 μϵ, outperforming the graphite-only mixture (10G0CMF) by 480%, 473%, and 86%, respectively. This mixture was subsequently used as the self-sensing node to be integrated into cementitious beam specimens. Three different embedded electrode designs were studied, consisting of 1, 2, and 3 zig-zag strip lines. These electrodes were 3D printed at the center of the beams, and performance was experimentally evaluated under quasi-static and dynamic flexural loading conditions.

Results showed that all embedded configurations provided linear and reliable strain-sensing responses ($R^{2}>0.97$), with the three-strip design yielding the highest average static and dynamic gauge factors of 669 and 630, respectively, and the two-strip configuration exhibited the highest signal quality, with SNR_stat_ and SNR_dyn_ of 9.50 dB and 10.8 dB, respectively. The one-strip configuration demonstrated the lowest strain resolution (34 μϵ). These results show that the electrode configuration can play an important role in sensing performance and that more work is needed to draw conclusions on such an effect.

Overall, this study demonstrates the feasibility of embedding localized self-sensing layers within 3D-printed structural elements using cementitious composites functionalized with carbon-based fillers. The developed approach offers a scalable and durable method for integrating strain-sensing capabilities into printed cementitious components, with possible applications to the condition assessment of 3D-printed cementitious components and structures. It also offers a solution to creating self-sensing concretes that are otherwise difficult to fabricate due to the presence of aggregates. Left to future work is the systematical evaluation of the influence of nozzle size, stand-off distance, layer bonding, and printing interruptions on both print quality and sensing performance, in an effort to empower hybrid manufacturing capabilities.

## Figures and Tables

**Table 1:** Material properties for the Fisher Chemical, APS 7–11 μm, 99% G powder and SGL milled and chopped CMF [80].

Material Properties	Units	G Powder	MCMF	CCMF
density	g/cm^3^	1.06	1.80	1.80
mean fiber length	μm	-	150	6000
filament diameter	μm	37	7	7
tensile strength	GPa	-	4.0	4.0
tensile modulus	GPa	-	240	240
elongation at break	%	-	1.7	1.7
single filament ρ	μΩm	40	15	15
bulk density	g/L	800	250	-
sizing type	-	unsized	unsized	glycerin
sizing mass content	%	-	-	4.0

**Table 2:** Material properties of mixture components. Density values correspond to bulk density for water and cement, and true density for G and CMF, based on manufacturer datasheets. Conductivity values are reported as specific conductivity in S/m. Aspect ratios are taken from the literature [77].

Material	Density (g/cm^3^)	Conductivity (S/m)	Aspect Ratio (-)
water	1.0	5 × 10^−2^	N/A
cement (powder)	1.5	0.9–1.5 × 10^−6^	N/A
G (powder)	2.2	2–3 × 10^3^	≈12
MCMF	1.8	4.3 × 10^4^	≈80
CCMF	1.8	6.5 × 10^4^	≈1000

**Table 3:** Mixture proportions for each set of specimens.

Mixture Type	Cement (g)	G (g)	G/c (%)	MCMF (g)	MCMF/c (%)	CCMF (g)	CCMF/c (%)	w/c (%)
0G0CMF	120	-	-	-	-	-	-	0.360
5G0CMF	120	6	-	-	-	-	-	0.483
10G0CMF	120	12	-	-	-	-	-	0.583
5G125M125CCMF	120	6	5	0.15	0.125	0.15	0.125	0.483
5G250M125CCMF	120	6	5	0.3	0.250	0.15	0.125	0.483
5G375M125CCMF	120	6	5	0.45	0.375	0.15	0.125	0.483
5G500M125CCMF	120	6	5	0.6	0.500	0.15	0.125	0.483
10G125M167CCMF	120	12	10	0.15	0.125	0.2	0.167	0.583
10G250M167CCMF	120	12	10	0.3	0.250	0.2	0.167	0.583
10G375M167CCMF	120	12	10	0.45	0.375	0.2	0.167	0.583
10G500M167CCMF	120	12	10	0.6	0.500	0.2	0.167	0.583

**Table 4:** Averaged strain and stress sensing performance of each mix design.

Mix Design	$\lambda_{\mathrm{dyn}}$ (-)	$\sigma_{\lambda }$ (-)	$\Delta_{\lambda }$ (%)	SNR (dB)	$\Delta_{\mathrm{SNR}}$ (%)	MAE (μϵ)	$\Delta_{\mathrm{MAE}}$ (%)
0G0CMF	22	2.4	-	1.83	-	7562	-
5G0CMF	95	7.0	-	3.35	-	5251	-
10G0CMF	181	13.52	193	7.49	124	3219	38
5G125M125CCMF	301	8.16	217	9.93	196	2251	57
5G250M125CCMF	331	11.44	248	10.85	224	2638	49
5G375M125CCMF	356	24.99	274	12.65	277	1382	74
5G500M125CCMF	382	9.74	302	16.22	384	3534	33
10G125M167CCMF	387	13.56	307	11.68	249	1293	75
10G250M167CCMF	550	12.25	480	19.19	473	721	86
10G375M167CCMF	401	9.09	322	15.80	372	1639	69
10G500M167CCMF	351	12.32	269	14.52	333	1004	81

**Table 5:** Averaged strain and stress sensing performance of each strip configuration.

Mix Design	SNR_stat_ (dB)	SNR_dyn_ (dB)	Δ_SNR_ (%)	MAE (μϵ)	*R*^2^ (-)	$\lambda_{\mathrm{stat}}$ (-)	$\lambda_{\mathrm{dyn}}$ (-)	95% CI		$\sigma_{\mathrm{res}}$ (μϵ)
								-FCR	res ( μϵ)	
1-strip—S1	7.71	8.06	4.54	212	0.98	664	621	0.017	28	-
1-strip—S2	8.95	9.10	1.68	224	0.97	629	579	0.020	35	-
1-strip—S3	9.07	10.4	14.88	249	0.99	601	551	0.018	39	-
average	8.58	9.19	7.04	228	0.98	631	583	0.018	34	5.56
2-strip—S1	10.02	11.4	13.67	219	0.97	596	542	0.018	40	-
2-strip—S2	9.83	10.6	7.93	132	0.98	581	509	0.022	44	-
2-strip—S3	8.65	9.58	10.75	244	0.97	536	481	0.021	50	-
average	9.50	10.74	14.3	198	0.98	571	510	0.020	44	5.03
3-strip—S1	8.08	8.72	7.92	106	0.98	686	652	0.025	39	-
3-strip—S2	7.34	8.21	11.85	242	0.97	701	658	0.028	43	-
3-strip—S3	8.51	9.82	15.39	206	0.96	621	580	0.029	58	-
average	7.98	8.91	11.7	185	0.97	669	630	0.027	46	10.0

## Data Availability

The data are available in a publicly accessible repository at the following link: https://www.dropbox.com/scl/fo/440tbmaglu1w3lk2mi4x7/AEZvLmC_Mga1EDj-jiVXOpU?rlkey=8pozofdouoknmh5gd0b399qbi&st=66rnugbm&dl=0 (accessed on 25 September 2025).

## Abbreviations

The following abbreviations are used in this manuscript:

SHM	Structural Health Monitoring
3DP	3D Printing
G	Graphite
MCMF	Miller Carbon Microfiber
CCMF	Chopped Carbon Microfiber
CMF	Carbon Microfiber
FCR	Fractional Change in Resistivity
SNR	Signal-to-Noise Ratio
MAE	Mean Absolute Error
DAQ	Data Acquisition System
PE	Polyethylene
PSD	Power Spectral Densities
CI	Confidence Interval
